# Effect of tetracycline treatment regimens on antibiotic resistance gene selection over time in nursery pigs

**DOI:** 10.1186/s12866-019-1619-z

**Published:** 2019-12-02

**Authors:** Kaare Græsbøll, Inge Larsen, Julie Clasen, Anna Camilla Birkegård, Jens Peter Nielsen, Lasse Engbo Christiansen, John Elmerdahl Olsen, Øystein Angen, Anders Folkesson

**Affiliations:** 1 DTU Compute, Technical University of Denmark, Kongens Lyngby, Denmark; 2 Department of Veterinary and Animal Sciences, University of Copenhagen, Copenhagen, Denmark; 3 DTU Vet. Technical University of Denmark, Kongens Lyngby, Denmark; 4 Present address: SSI, Copenhagen, Denmark; 5 DTU BioEngineering, Technical University of Denmark, Kongens Lyngby, Denmark

**Keywords:** Oxytetracycline, Resistance genes, qPCR, Nursery pigs

## Abstract

**Background:**

The majority of antimicrobials given during the production of pigs are given to nursery pigs. The influence of antimicrobial use on the levels of antimicrobial resistant (AMR) genes is important to quantify to be able to assess the impact of resistance on the food chain and risk to human and animal health.

**Results:**

This study investigated the response on the levels of nine AMR genes to five different treatment strategies with oxytetracycline, and the dynamics of gene abundance over time by following 1167 pigs from five different farms in Denmark.

The results showed no significant difference between treatments and an increase in abundance for the efflux pump encoding *tet(A)* gene and the genes encoding the ribosomal protection proteins tet*(O)* and *tet(W)* tetracycline resistant genes following treatment, while *tet(M)* showed no response to treatment. However, it was also observed that the levels of *tet(O)*, *tet(W)*, and *ermB* in some farms would drift more over time compared to a single treatment-course with antibiotic.

**Conclusion:**

This study underlines the large variation in AMR levels under natural conditions and the need for increased investigation of the complex interactions of antimicrobial treatment and other environmental and managerial practices in swine production on AMR gene abundance.

## Background

Antimicrobial resistance (AMR) in bacteria is a growing global health concern that threatens to decrease the effectiveness of infectious disease treatment both in humans and animals [1–5]. Resistance towards antibiotics is an ancient characteristic that presumably co-evolved with the evolution of antibiotic producing organisms and is widespread in nature [6–10]. The intensive use of antibiotics in society in general, and in the food production systems in particular, may increase the chance of transmission of antibiotic resistant microorganisms to humans from livestock or the environment through the consumption of food products of animal origin or through water and soil [11–14].

The effect of antimicrobial use in food production on AMR in humans has been difficult to quantify [15–20] and routes and the rates of AMR transmission are not well characterized [21]. Moreover, the impact of AMR on animal health and food production is even less elucidated. A reason for this could be the reliance on culture based methods for detection of pathogens and indicator organisms, since such methods are unable to quantify the AMR gene prevalence in the commensal microflora [21]. Recent technological developments in molecular biology, however, have increased our ability to investigate and quantify the gene content of microbial communities without cultivation. This has enabled us to study to what extent the normal flora is affected by the use of antimicrobials, and to detail the molecular ecology of AMR genes [21–26].

Gastrointestinal bacterial infection is a common disease in many animal production systems and an important incentive for antimicrobial treatment [27–30]. Oral administration of antimicrobials is widely used to control such infections in the pig industry [31–33]. In Denmark, tetracycline has been the most commonly used antimicrobial for oral treatment of gastrointestinal disorders in pigs, such as *Lawsonia intracellularis* (*L.intracellularis*) infections [34].

The world organisation for animal health (OIE) recommends clinical trials to evaluate the validity of therapeutic indications and dosage regimens, to protect both animal and human health as well as the environment [35]. The effect of oxytetracycline treatment on faecal shedding of *L. intracellularis* and weight gain in nursery pigs suffering from diarrhoea has recently been investigated in a clinical field trial in Denmark, where the effect of different dosage regimens and administration routes were studied [33, 36]. Here, we study the impact of these different treatment regimens on the prevalence and levels of nine AMR genes (*ermB, ermF, sulI, sulII, tet(A)*, *tet(B)*, *tet*(M), *te*t(O) and *tet*(W)) which are frequently present in pigs on Danish pig farms [37, 38] The aim of the present study was to determine the effect of five different oxytetracycline treatment regimens on a selection of AMR genes in the normal flora in nursery pigs in a randomized clinical field trial.

## Results

### Sample collection

A total of five farms were included in the study, and samples were collected from a total of 1167 ear marked pigs. 240 pigs received batch treatment with High Dose (HD), 241 with Normal Dose (ND) and 224 with Low Dose (LD) of oxytetracycline (OTC). 241 pigs were included in the pen wise treatment group (PW), and 221 in the injection treatment group (IM). Of the 241 pigs in the PW group, 204 received treatment with OTC, and of the 221 pigs in the IM group, 124 received treatment with OTC. The pigs where sampled before treatment with oxytetracycline (T1), two days after end of treatment (T2), and when exiting the nursery stable (T3). Each farm was assigned a stratified random treatment group in sequential batches, where an entire section would be treated in the same way. After a completed batch a new batch would randomly be assigned to the same or a different treatment. Typically, each farm were assigned to every treatment regime three times (ranging from two to four).

### Enrichment of tetracycline resistance genes after tetracycline treatment

We analysed the relative abundance and prevalence of nine individual antibiotic resistance genes in the samples collected at three time points from the five different treatment groups; *ermB, ermF,* that confers macrolide resistance; *sulI, sulII* conferring resistance towards sulphonamides and *tet(A), tet(B), tet(M), tet(O),* and *tet(W)* that confers increased resistance towards tetracycline. Five genes *ermB, ermF*, *tet(M), tet(O),* and *tet(W)* were always present and was quantified relative to the number of 16 s RNA genes in the same sample, while four genes, *sulI*, *sulII*, *tet(A)*, and *tet(B)*, were only sporadically present and were analysed based on prevalence. Relative quantities and prevalence of genes are presented in Additional file 1: Figure S1, Additional file 2: Figure S2, Additional file 3: Figure S3, Additional file 4: Figure S4, Additional file 5: Figure S5, Additional file 6: Figure S6, Additional file 7: Figure S7.

**Fig. 1:**
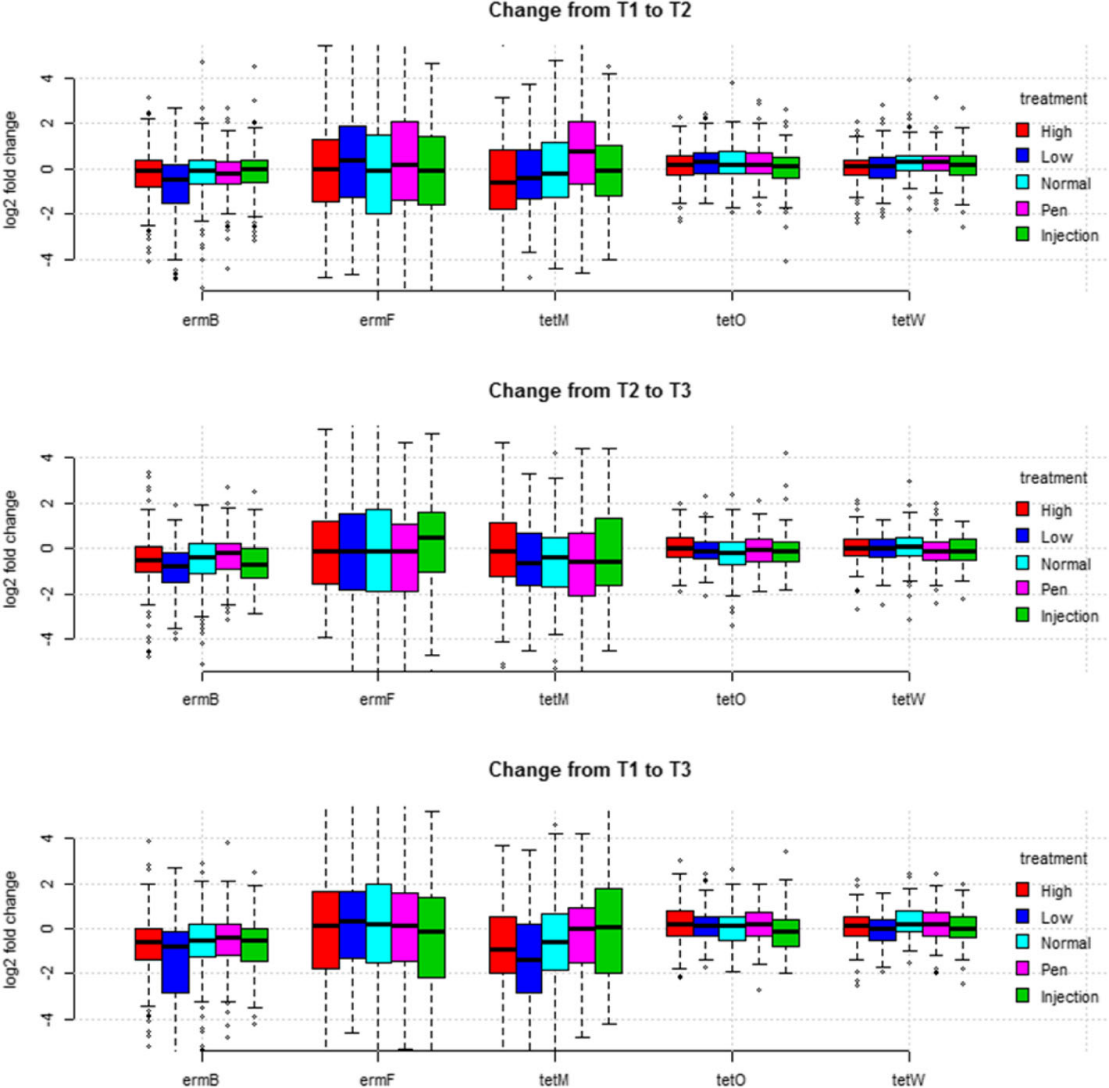
Fold change of the relative quantity of five resistance genes following different treatment strategies. Fold change is displayed from before treatment to two days after treatment (T1 to T2, top), from after treatment to exit from the weaner stable (T2 to T3, middle), and from start of treatment to exit from the weaner stable (T1 to T3, bottom). The y-axis is on log2 scale meaning that a value of one equals that a gene is present in twice the amount relative to the 16S gene at the later time compared to the prior time, a value of zero is no change, and minus one is half the prior level. Boxes represent the interquartile range, whiskers represent the range of the data limited to 1.5 interquartile range

Figure 1 shows the changes in levels of genes from before treatment to after treatment (T1 to T2), from after treatment to exit of the nursery unit (T2 to T3), and over the entire stay in the nursery unit (T1 to T3). Generally, two genes, *ermF* and *tetM* showed the largest variation in how pigs responded to the different treatments, however, no differences were observed in changes for any of the five genes analysed depending on the treatment (dose variation and variation in application).

**Fig. 2:**
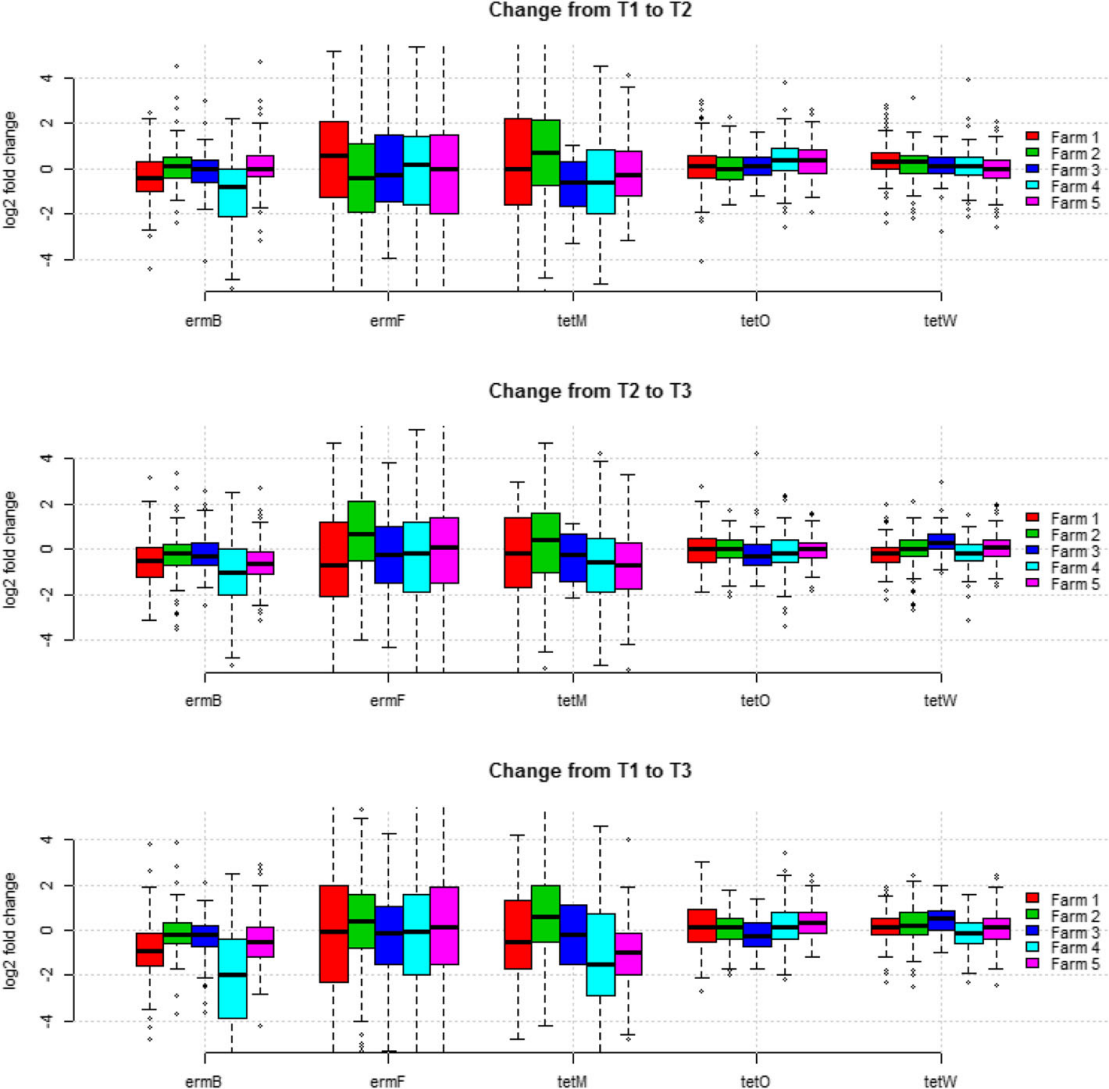
Fold change of the relative quantity of five resistance genes aggregated on the five participating farms. Fold change is displayed from before treatment to two days after treatment (T1 to T2, top), from after treatment to exit from the wiener stable (T2 to T3, middle), and from start of treatment to exit from the wiener stable (T1 to T3, bottom). The y-axis is on log2 scale meaning that a value of one equals a that a gene is present in twice the amount relative to the 16 s gene at the later time compared to the prior time, a value of zero is no change, and minus one is half the prior level. Boxes represent the interquartile range, whiskers represent the range of the data limited to 1.5 interquartile range

Furthermore, all pigs originating from one farm were aggregated to visually inspect for changes of each gene in the five different farms (Fig. 2). Again, *ermF* and *tetM* showed the largest variation. As there was generally large variation in the degree of changes within and between the farms (Fig. 2) univariate statistics on the changes following different treatments, as shown in Fig. 1, may not be appropriate, and is therefore not reported.

### Test including all variables affecting gene abundance

To account for the impact of both farm and treatment, a mixed effect model including the random effect of batch was employed for all genes on all combinations of times (T1 to T3, T1 to T2, and T2 to T3). All tests had farm as a significant factor. After employing backwards elimination with an alpha level of 0.05, the model for *tet*(W) from T1 to T3 also had treatment strategy as a significant effect, but when applying a Bonferroni correction for multiple comparisons (alpha = 0.05/4, four tests are done on each ΔΔC_q_ per gene per time pair), this effect disappeared for *tet*(W). Thus, the multivariant analysis confirmed the preliminary indication from the univariate visualisation (Fig. 1) that treatment strategy did not affect the exit levels of the quantifiable genes (T3 compared to T1).

For prevalence of genes, which was calculated per batch and employing backwards elimination on a model including farm, time, and treatment effects, it was again found that farm was a significant factor, and that a HD treatment increased *tet*(A) and *tet*(B) to higher levels between T1 and T2 compared to the other treatment regimens (*p* = 0.001/0.03). However, only *tet*(A) was significant when taking repeated testing into account.

The overall level of some genes also changes over the time in the nursery (from T1 to T3). For *ermB* a reduction of 48% (*p* = 0.004) was seen, and for *tet*(W) an increase of 27% (*P* = 0.01) was seen. For *tet*(A), and overall reduction of 19% points (*p* = 0.01), *sulI* a reduction of 7% points (*p* = 0.03), and *sulII* a reduction of 12% points (*p* < 0.0002) was observed.

In the PW and IM groups, not all animals were treated. It was therefore possible to test whether treatment with oxytetracycline had any effects on gene levels. For *tet*(O) and *tet*(W), the relative change in gene copies was significantly higher from T1 to T2 and from T1 to T3 in treated pigs, also when employing a Bonferroni correction. The treatment increased the levels of *tet*(O) from T1 to T2 with 27% (*p* = 0.001), *tet*(W) from T1 to T2 with 22% (*p* < 0.001); *tet*(O) from T1 to T3 with 24%, (*p* = 0.009) and *tet*(W) from T1 to T3 with 17% (*p* = 0.006). There were too few whole batches pigs receiving no treatment to test the effect on prevalence of the remaining genes.

### Time effects antibiotic resistance gene composition at farm level

To evaluate the change of AMR gene levels over time longer than the single batch, the levels of *ermB*, *tet*(O), and *tet*(W) at start of treatments, right after treatment and at the exit from the nursery unit, irrespective of treatment, were plotted over the full time of trial (Additional file 7: Figure S7, Additional file 8: Figure S8, Additional file 9: Figure S9, Additional file 10: Figure S10, Additional file 11: Figure S11). All genes showed drift in the level of abundance on one or more farms over the course of the trial. For comparison, we compared T1 in the first three and the last three batches including at each farm: On Farms 2, 4, and 5 *ermB* was significantly reduced with 37, 72, and 70% (all *p* < 0.001). On Farms 1 and 5 *tet(O)* decreased with 72, and 29% (both *p* < 0.001), while farm 2 had an increase of 117% (*p* < 0.001). On Farms 1, 3, and 4 there was significant decrease of *tet(W)* of 21, 16, and 17% (*p* = 0.003, *p* = 0.020, *p* = 0.004), while there was an increase at farm 2 of 21% (*p* = 0.05) (Additional file 8: Figure S8, Additional file 9: Figure S9, Additional file 10: Figure S10, Additional file 11: Figure S11, Additional file 12: Figure S12).

Prevalence of AMR genes *tet*(A), *tet*(B), *sulI,* and *sulII* was also plotted over time (Additional file 13: Figure S13, Additional file 14: Figure S14, Additional file 15: Figure S15, Additional file 16: Figure S16, Additional file 17: Figure S17). These plots also showed changes over time. The behaviour seems more erratic than the quantifiable genes, likely because many of these genes were present in level close to the limit of detection, and so the variation in the results becomes high. Furthermore, when aggregating on batch the number of data points decrease so low *p*-values become rarer, and so there were no significant p-values when correcting for repeated measurements. Though, the closest to significance where the increase of *tet*(A) over time (at T1) in farms 2 and 5.

### Pigs from the same farm have more similar resistance gene composition than pigs from different farms

**Fig. 3:**
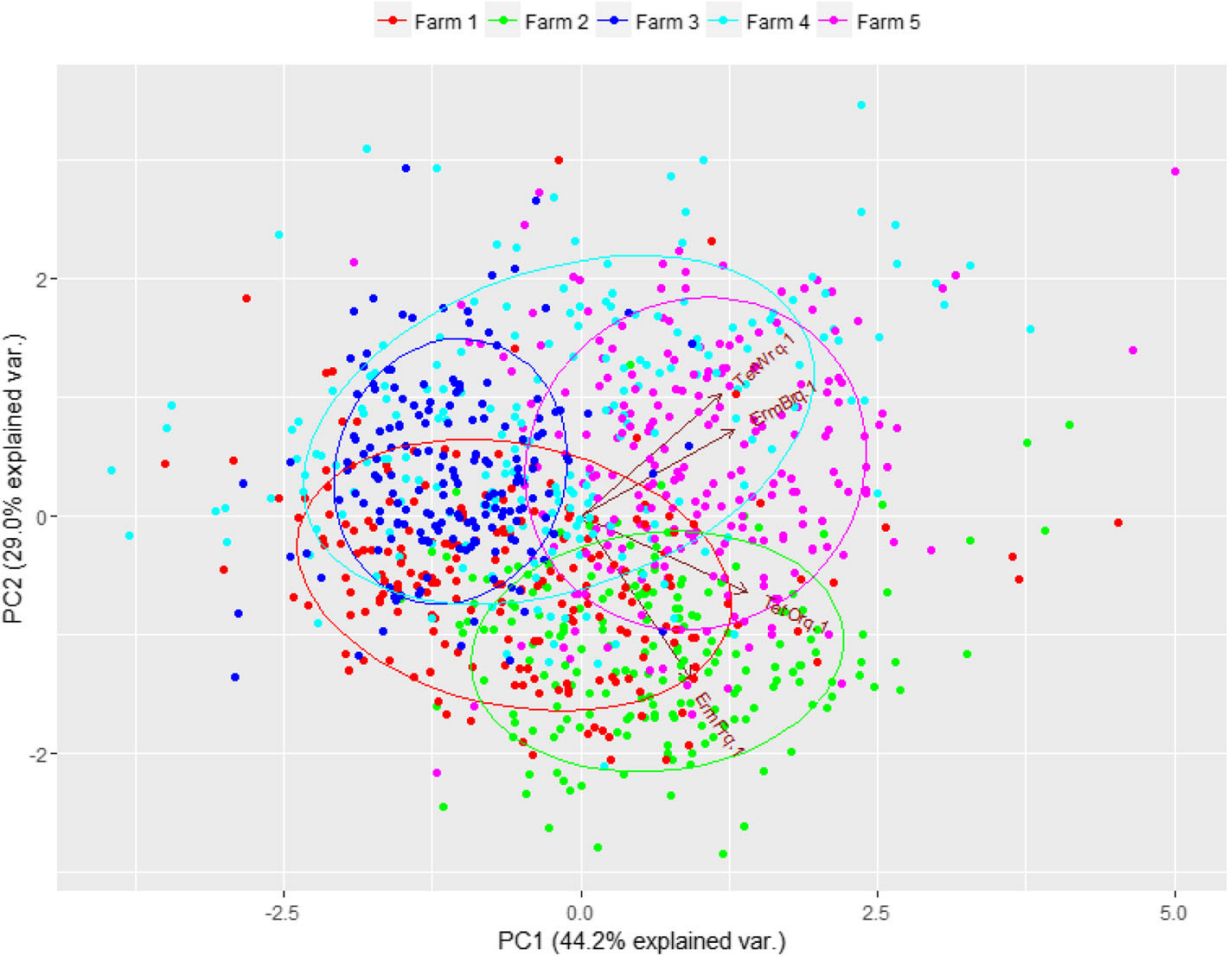
Principal component analysis of gene composition of pigs at the five participating farms. Values used were Relative quantities of *tet(O)*, *tet(W)*, *ermB*, and *ermF* compared to *16S* prior to treatment at T1. Ellipses are normal data ellipses for each farm. Data was scaled and centred

**Fig. 4:**
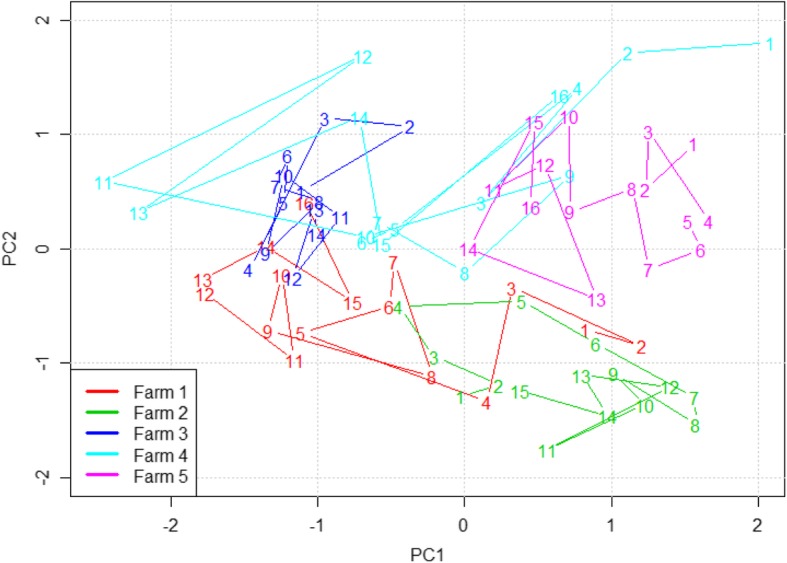
Principal component analysis of gene composition of pigs at the five participating farms. Numbers are chronologically per farm and indicate the centre of each batch prior to treatment (T1). The PCA transformation is identical to Fig. 3

A principal component analysis showed that the pigs in farms 2 and 3 had very similar composition of resistance genes (small clusters), while farm 4 had pigs with large variation in gene composition and farms 1 and 5 had medium variation (Fig. 3). When aggregating pigs per batch at T1 (Fig. 4) all farms except farm 4 showed a tendency that chronologically appearing batches clustered close to each other.

## Discussion

The purpose of this study was to investigate how treatment dose and regimen with oxytetracycline affected the levels of nine AMR genes that are commonly found in the microflora of nursery pigs (*tet*(A),(B), (M),(O),(W)) [39–44], *erm* (B) and (F) [44–46], *sulI* and *sulII* [38, 47, 48]. These included genes that were directly selected by the treatment (the *tet* genes), and genes that were not expected to be selected by the treatment (the *erm* and *sul* genes). Additional inclusion criteria were resistance determinants towards antibiotic classes frequently used of the in Danish pig production, and to be feasible to design qPCR assays having the same temperature profile. The tetracycline resistance *tet* genes are broadly disseminated and have been found in a large number Gram negative, Gram positive, aerobic and anaerobic bacteria, where they often are harboured on horizontally transferred genetic elements [49]. The *erm* genes are associated with transposons also widely distributed in many bacterial genera and species where the tetracycline resistance gene *tet*(Q) is often linked to *erm*(F) and *tet*(M) to *erm*(B) [50, 51]. The study enabled a unique incorporation of variation between farms and pigs in the evaluation of the effect of different oxytetracycline treatments.

A large number of studies have investigated the effect of antibiotic therapy on antibiotic resistance. The majority of these studies have concerned phenotypic resistance in coliform indicator bacteria [52–55]. Recent developments in DNA based molecular technologies, however, have enabled metagenomic surveillance of resistance determinants in microbial communities [21, 24, 56–58] which allow a much more comprehensive sampling of the AMR gene prevalence [59]. Here we study the effects of different oxytetracycline treatment strategies on the levels of nine resistance genes under field conditions using high capacity qPCR.

There was a significant decrease in the relative levels of three out of four non-tet genes from T1 to T3 (Figs. S12-S17). However, the use of oxytetracycline significantly increased the relative levels of the four tetracycline resistance genes; *tet*(A), *tet*(B), *tet*(O), and *tet*(W) (Figs. 1 and 2, S12-S17) during treatment from T1 to T2, followed by a slight decline after treatment from T2 to T3, but to an overall increasing effect. *Tet*(O) and *tet*(W) encode closely related tetracycline ribosomal protection proteins and are ubiquitously found in gut and soil microbiomes [21, 22, 38, 60, 61]. *tet*(A), and *tet*(B), encodes proteins that belong to the major facilitator superfamily (MFS) which export tetracycline from the cell [62]. This effect of treatment correlates well with the results of a phenotypic investigation of tetracycline resistance in coliform bacteria from the same clinical trial published elsewhere [55]. However, the increase of abundance was not generally dose dependent (except for tet(A)) or dependent on whether individual, pen-wise or batch treatment regimens were used, and the overall amount of all the AMR genes included in the study decreased over time in the nursery period, which confirms previous observations [63]. Moreover, the abundance of *tet*(M), which also encodes a Group-1 ribosomal protection protein of same protein family as *tet*(O) and *tet*(W), did not increase significantly during the trial, but followed the general decreasing trend shown by genes not associated with tetracycline resistance (Fig. 1 and Additional file 15: Figure S15) [64]. The tetracycline resistance genes investigated in the current study are examples of very abundant genes with widespread distribution, and they can be situated both on the chromosome and on horizontally transferred vectors [64, 65]. Differences in genetic context may be a contributing factor in the differential response to oxytetracycline treatment that was observed for *tet*(O) and *tet*(W) compared to *tet*(M). A gene encoded on a plasmid or other mobile genetic elements would be expected to have a more dynamic response towards selection due to gene amplification and changes in copy number. It has been recently shown that *Yersinia pseudotuberculosis* regulate plasmid copy number in response to environmental cues to increase expression of plasmid borne virulence factors and a similar regulatory mechanisms could be involved in the dynamic regulation of AMR gene copy numbers of resistance genes carried on plasmids [66]. However, this mechanism remains to be shown to be important in an AMR context. Moreover, a resistance gene situated on a transferable element can quickly transfer to a new host while a chromosomally encoded gene mainly increases in numbers by following the host proportion in the community. This possibility highlights a complication of using relative values of gene abundance in whole community samples to study gene abundance, because a gene can paradoxically increase in prevalence within the host’s bacterial population, but decrease in relative abundance due to large changes in the microbiome composition. This could have implications for surveillance using DNA based methods, because zoonotic pathogens, which frequently have small populations, could have a proportionally high resistance level but a low over all relative prevalence level. Oxytetracycline dose and use of batch treatment as opposed to pen wise and single animal injection treatment have previously been shown to improve the clinical efficacy of treatment of *Lawsonia intracellularis* infection [33, 36]. Therefore, batch treatment may in some situations be considered a safe and effective treatment regimen for intestinal infection in pigs. In order to reduce AMR a strategy for reducing the number of treatments rather than the treatment regimens (batch, penwise, injection) should be preferred [64].

There was a large variation in the amount of AMR genes between the farms included in the clinical trial both in relative gene levels and change over time, (Fig. 2 and Additional file 7: Figure S7, Additional file 8: Figure S8, Additional file 9: Figure S9, Additional file 10: Figure S10, and Additional file 11: Figure S11) and pigs from the same farm had a more similar resistance gene composition than pigs from different farms (Fig. 3). AMR gene levels also fluctuated significantly over time in the individual farms indicating that batch resistance-gene composition play an important role in the overall AMR gene abundance (Fig. 4). AMR is influenced by factors such as animal husbandry, geographic location, and trade connections. Interestingly it was recently shown that the resistance composition of the sow’s bacterial flora strongly influenced the resistance gene prevalence in the offspring even though the piglets were moved to other farms [37]. This observation could explain the similarity between pigs on the same farm due to the increased probability that they have a similar origin and disease/treatment history. Furthermore, the different composition of genes on different farms (Figs. 3-4) may be an explanatory factor of the different response seen to treatment (Fig. 2).

When all factors were included in a mixed effect model, only *tet*(A) showed effect of treatment dose, and only *tet(O)* and *tet(W)* were significantly affected by oxytetracycline. That treatment but not treatment mode was significant is likely because the numbers of untreated pigs in the treatment groups IM and PW were low, and non-treated animals in the IM group resided inside the same pens as treated animals.

## Conclusion

This study exemplifies the complexity of AMR gene abundance in real life situations. Oxytetracycline resistance gene levels were slightly but significantly increased during treatment, but only for four out of nine resistance genes included in the study. The analysis also shows that non-treatment factors such as animal history and management procedures may strongly influence the resistance levels on the individual farm and that the effect of a single antibiotic treatment on resistance levels at the weaning stage may have a minor influence on the overall resistance gene abundance and prevalence. The study also shows the utility of using DNA based analysis methods that can cheaply and quickly analyse a large number of samples: however, the study also illustrates the large variability in gene levels under natural conditions, which complicates analysis of the population genetics and microbial ecology of resistance gene.

## Methods

### Clinical field trial

The randomized clinical field trial has previously been described in two studies measuring the effect of varying treatment-doses and treatment-routes on the effect of oxytetracycline (OTC) treatment against *L. intracellularis* induced diarrhoea [33, 36]. Briefly, five farms were selected based on sampling to represent a typical problem with *L. intracellularis* (LI) induced diarrhoea amongst the clients of two specialised veterinary pig practices [33, 36, 55]. Farms vaccinated against LI or high risk of acute medication were excluded. In each farm, 15 batches were included in the study after being weaned and followed until the end of the nursery period seven weeks after they were introduced into the nursery unit. A batch was defined as a group of nursery pigs all weaned at the same time, housed into the same large room, and sub-divided into smaller compartments, the pens. The number of pigs per batch ranged from 240 to 600 and the number of pens per batch ranged from 8 to 20 depending on which ‘room’ was used at the specific farm. In each batch, 15 animals, randomly distributed over pens, were selected as trial pigs [33, 36]. and all trial pigs were ear tagged with a unique ID as previously described in [33, 36].

Each batch was monitored once a week for outbreak of diarrhoea. The estimation of diarrhoea was done by trained veterinarians, responsible for initiating treatment. When an outbreak was diagnosed, one of five treatment protocols, each consisting of a treatment once a day for five days was used: Oral batch-treatment in water with a standard dose of 10 mg/kg OTC (Terramycin®Vet. 20%, Orion Pharma) (ND), oral batch- treatment in water with a high dose of 20 mg/kg OTC (HD); oral batch-treatment in water with a low dose of 5 mg/kg OTC (LD), oral pen-wise treatment in water with a standard dose of 10 mg/kg OTC (PW) or individual intra muscular treatment with a standard dose of 10 mg/kg OTC (IM). For batch treatment, an outbreak was defined as more than 0.5 deposits of stool on average per pen floor or more than 25% of the pigs with clinical diarrhoea. Pen-wise treatment was initiated when the outbreak criteria was fulfilled for a pen (a smaller subset of the batch housed in the same room), while individual treatment was initiated when animals showed typical signs of diarrhoea (loose to watery faeces or poor body score). For batch and pen-wise treatment, it was ensured that the medicine was consumed within 24 h. Each protocol was repeated 3 times on each farm, and the order of treatment protocols was chosen at random to avoid bias.

### Sampling

Faecal samples were collected as previously described by Græsbøll et al. (2017) [55]: Briefly, samples were collected from all trial pigs between October 2011 and April 2013, either at defecation or per rectum. Samples were collected from all pigs at three time points: Time point 1 (T1) was the first day of treatment; immediately before antibiotic administration, Time point 2 (T2) was two days after the end of treatment, and Time point 3 (T3) was when pigs were moved from the nursery unit to finisher stables. The clinical study was designed to be able to measure differences in production parameters during the time in the nursery unit, most importantly growth rate. Samples were stored in 40 ml containers and shipped to the laboratory in cooled boxes.

### DNA extraction

Total DNA was extracted from the samples, using the Maxwell® 16 LEV Blood DNA Kit (Promega Corporation, Madison, WI, USA) as described by Clasen et al. [25]. In short, samples were homogenized in a cell and tissue disruptor (Tissuelyser II, Qiagen, Hilden, Germany) with 5 mm stainless steel beads (Qiagen, Hilden, Germany) and bead-beated for 2 min in a 25 mg/ml lysozyme (Sigma-Aldrich, Denmark A/S)) buffer. Samples were then transferred to Maxwell® extraction kit cartridges and DNA was extracted in according to manufacturer’s instructions. DNA concentrations were measured and purity were assessed by the 260/280 nm-ratio using the NanoDropND-1000 spectrophotometer (NanoDrop Technologies Inc., Wilmington, DE, USA). Samples were diluted to 40 ng/μl in nuclease free water (Qiagen, Hilden, Germany) and stored at − 20 °C until further processing.

### Real-time qPCR primers

qPCR amplifications for the quantification of *tet(A), tet(B), tet(M), tet(O), and tet(W)*, representing genes that were directly selectable by the tetracycline treatment, and *ermB, ermF, sulI and sulII* representing genes encoding resistances that were unrelated to the tetracycline treatment*,* and 16S rRNA-gene as an approximation for the number of bacteria in total DNA extracted from pig faecal samples were performed using the Fluidigm HD Biomark system (Fluidigm Corporation, South San Francisco, CA, USA). Levels of the AMR genes, were quantified using the high-capacity qPCR chip Gene Expression 192 × 24 (Fluidigm Corporation, South San Francisco, CA, USA) with two technical replicates using 16S as reference gene as previously described [25]. Primers used are listed in Additional file 18: Table S1).

### Statistics

Quantifiable genes was put on the form of relative abundance (ΔC_q_ = (C_q, 16s_)_Tx_ - (C_q, gene_)_Tx_) or relative fold change (ΔΔC_q_ = (C_q, 16s_-C_q, gene_)_Ty_ - (C_q, 16s_-C_q, gene_)_Tx_), were Tx and Ty represent different time points. Test for significant change in relative quantities (RQ = ΔΔC_q_) between time points in treatment strategies or on farms were performed using a two-sided t-test with the null hypotheses being no change. To determine the effect of treatment regime including all factors ΔΔC_q_ values were also tested using farm and treatment strategy as fixed effects and batch as random effect. Furthermore, to determine the effect of treatment of the individual pig, ΔΔC_q_ values were tested using farm and treatment as fixed effects and batch as random effect. Changes reported in percentages are equivalent to (2^ΔΔCq^ – 1)*100%. In case of genes where low abundance resulted in difficulty of quantifying, the genes were aggregated to prevalence per batch and tested using linear regression using farm, time, farm:time, and treatment:time as fixed effects.

Tests using fixed and random effects were performed using lmer from the package lme4, and backwards elimination was performed using step from the package lmerTest. Linear regression was performed using the function glm with a Gaussian family. Principal component analysis was performed on the relative quantities using prcomp with scaling and centering options enabled. All tests were performed in R version > 3.2 (R Core Team, 2015).

## Supplementary information

**Additional file 1: Figure S1.** Relative quantities (RQ) of *tet(O)*, *tet(W)*, and *ermB* to *16S* of all batches chronologically onfarm 1. Top graph is values prior to treatment (T1), middle graph values two days after treatment (T2), and bottom graph is values at exit from nursery unit (T3). Dots are median values of each batch. Lines are smoothed values of the data points. Coloured areas are smoothed areas of the interquartile range.

**Additional file 2: Figure S2.** Relative quantities (RQ) of *tet(O)*, *tet(W)*, and *ermB* to *16S* of all batches chronologically farm 2. Top graph is values prior to treatment (T1), middle graph values two days after treatment (T2), and bottom graph is values at exit from nursery unit (T3). Dots are median values of each batch. Lines are smoothed values of the data points. Coloured areas are smoothed areas of the interquartile range.

**Additional file 3: Figure S3.** Relative quantities (RQ) of *tet(O)*, *tet(W)*, and *ermB* to *16S* of all batches chronologically on farm 3. Top graph is values prior to treatment (T1), middle graph values two days after treatment (T2), and bottom graph is values at exit from nursery unit (T3). Dots are median values of each batch. Lines are smoothed values of the data points. Coloured areas are smoothed areas of the interquartile range.

**Additional file 4: Figure S4.** Relative quantities (RQ) of *tet(O)*, *tet(W)*, and *ermB* to *16S* of all batches chronologically on farm 4. Top graph is values prior to treatment (T1), middle graph values two days after treatment (T2), and bottom graph is values at exit from nursery unit (T3). Dots are median values of each batch. Lines are smoothed values of the data points. Coloured areas are smoothed areas of the interquartile range.

**Additional file 5: Figure S5.** Relative quantities (RQ) of *tet(O)*, *tet(W)*, and *ermB* to *16S* of all batches chronologically on farm 5. Top graph is values prior to treatment (T1), middle graph values two days after treatment (T2), and bottom graph is values at exit from nursery unit (T3). Dots are median values of each batch. Lines are smoothed values of the data points. Coloured areas are smoothed areas of the interquartile range.

**Additional file 6: Figure S6.** Prevalence of *sulI, sulII, tet*(A), and *tet*(B). Stratified by sampling time and treatment.

**Additional file 7: Figure S7.** Prevalence of *sulI, sulII, tet*(A), and *tet*(B). Stratified by sampling time and farm.

**Additional file 8: Figure S8.** Relative quantities of *ermB* at times T1, T2 and T3. Stratified by treatment and farm.

**Additional file 9: Figure S9.** Relative quantities of *ermF* at times T1, T2 and T3. Stratified by treatment and farm.

**Additional file 10: Figure S10.** Relative quantities of *tet*M at times T1, T2 and T3. Stratified by treatment and farm.

**Additional file 11: Figure S11.** Relative quantities of *tet*(O) at times T1, T2 and T3. Stratified by treatment and farm.

**Additional file 12: Figure S12.** Relative quantities of *tet*(W) at times T1, T2 and T3. Stratified by treatment and farm.

**Additional file 13: Figure S13.** Prevalence of *tet(A)*, *tet(B)*, *sulI* and *sulII* of all batches chronologically on farm 1. Top graph is values prior to treatment (T1), middle graph values two days after treatment (T2), and bottom graph is values at exit from nursery unit (T3). Dots are median values of each batch. Lines are smoothed values of the data points.

**Additional file 14: Figure S14.** Prevalence of *tet(A)*, *tet(B)*, *sulI* and *sulII* of all batches chronologically on farm 2. Top graph is values prior to treatment (T1), middle graph values two days after treatment (T2), and bottom graph is values at exit from nursery unit (T3). Dots are median values of each batch. Lines are smoothed values of the data points.

**Additional file 15: Figure S15.** Prevalence of *tet(A)*, *tet(B)*, *sulI* and *sulII* of all batches chronologically on farm 3. Top graph is values prior to treatment (T1), middle graph values two days after treatment (T2), and bottom graph is values at exit from nursery unit (T3). Dots are median values of each batch. Lines are smoothed values of the data points.

**Additional file 16: Figure S16.** Prevalence of *tet(A)*, *tet(B)*, *sulI* and *sulII* of all batches chronologically on farm 4. Top graph is values prior to treatment (T1), middle graph values two days after treatment T2), and bottom graph is values at exit from nursery unit (T3). Dots are median values of each batch. Lines are smoothed values of the data points.

**Additional file 17: Figure S17.** Prevalence of *tet(A)*, *tet(B)*, *sulI* and *sulII* of all batches chronologically on farm 5. Top graph is values prior to treatment (T1), middle graph values two days after treatment (T2), and bottom graph is values at exit from nursery unit (T3). Dots are median values of each batch. Lines are smoothed values of the data points.

**Additional file 18: Table S1. **

## Data Availability

All data generated or analyzed during this study are included in this published article [and its supplementary information files].

## Abbreviations

AMR: Antimicrobial resistance
HD: High dose
IM: Injection treatment group
LD: Low dose
ND: Normal dose
OTC: Oxytetracyclin
PW: Pen wise treatment group
