# Supplementary material for: Effect of tetracycline treatment regimens on antibiotic resistance gene selection over time in nursery pigs
Source: BMC Microbiol. 2019 Dec 2;19:269. doi: 10.1186/s12866-019-1619-z (PMC6889206; doi:10.1186/s12866-019-1619-z)
Supplement: Supplementary file 3 — Additional file 3: Figure S3. Relative quantities (RQ) of tet(O), tet(W), and ermB to 16S of all batches chronologically on farm 3. Top graph is values prior to treatment (T1), middle graph values two days after treatment (T2), and bottom graph is values at exit from nursery unit (T3). Dots are median values of each batch. Lines are smoothed values of the data points. Coloured areas are smoothed areas of the interquartile range. [file 12866_2019_1619_MOESM3_ESM.pdf]

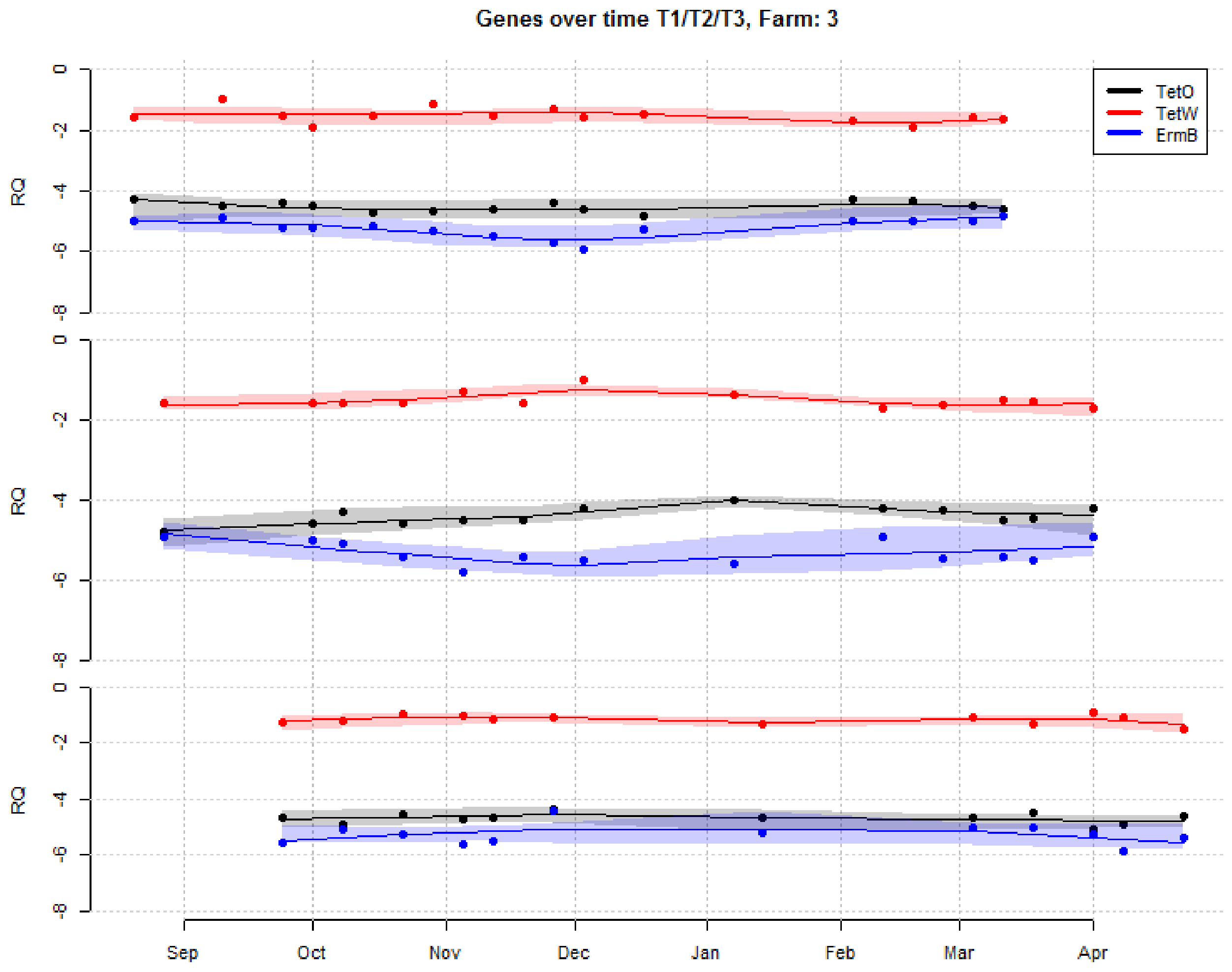

FIG S3: Relative quantities (RQ) of *tet(O)*, *tet(W)*, and *ermB* to *16S* of all batches chronologically on farm 3. Top graph is values prior to treatment (T1), middle graph values two days after treatment (T2), and bottom graph is values at exit from nursery unit (T3). Dots are median values of each batch. Lines are smoothed values of the data points. Coloured areas are smoothed areas of the interquartile range.
