# Supplementary figures and images for: Effect of tetracycline treatment regimens on antibiotic resistance gene selection over time in nursery pigs
Source: BMC Microbiol. 2019 Dec 2;19:269. doi: 10.1186/s12866-019-1619-z (PMC6889206; doi:10.1186/s12866-019-1619-z)

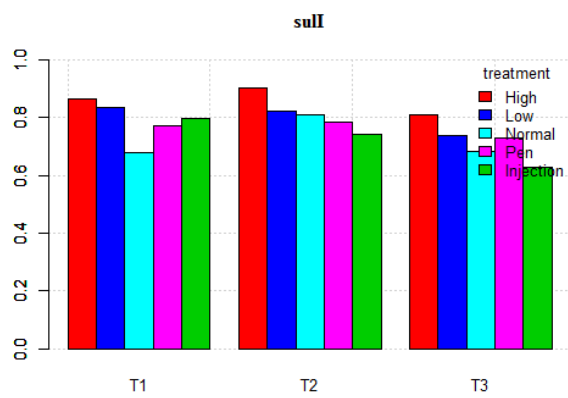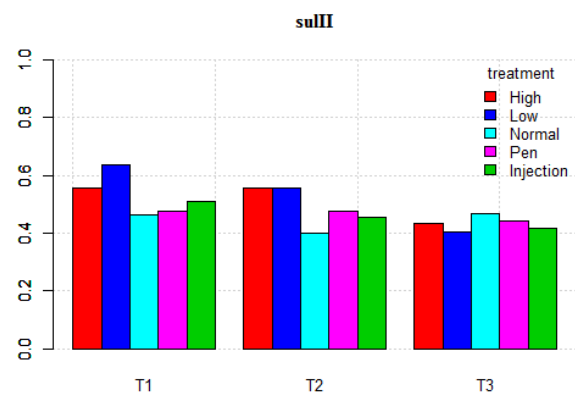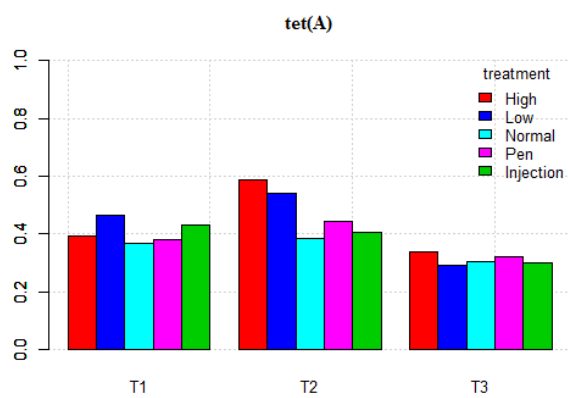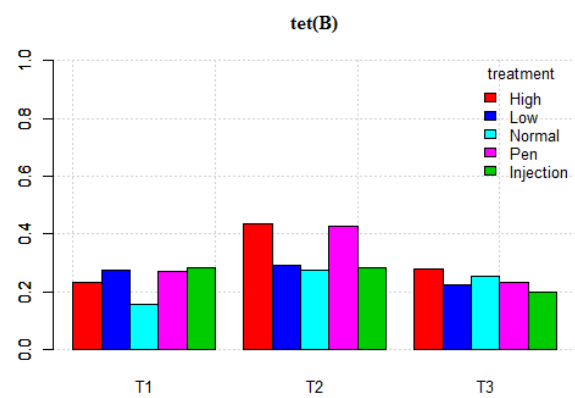

**FIG S6:** Prevalence of *sulI*, *sulIII*, *tet(A)*, and *tet(B)*. Stratified by sampling time and treatment.

Supplement: Supplementary file 6 — Additional file 6: Figure S6. Prevalence of sulI, sulII, tet(A), and tet(B). Stratified by sampling time and treatment. [file 12866_2019_1619_MOESM6_ESM.pdf]

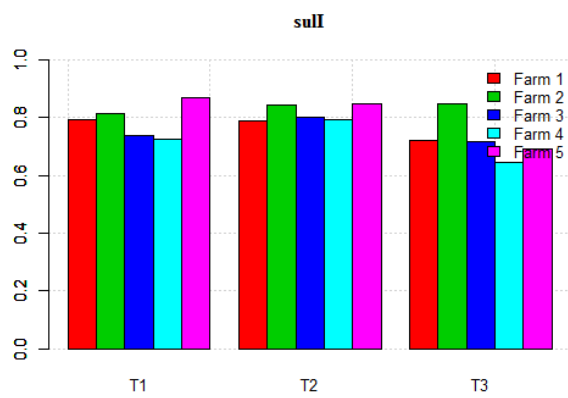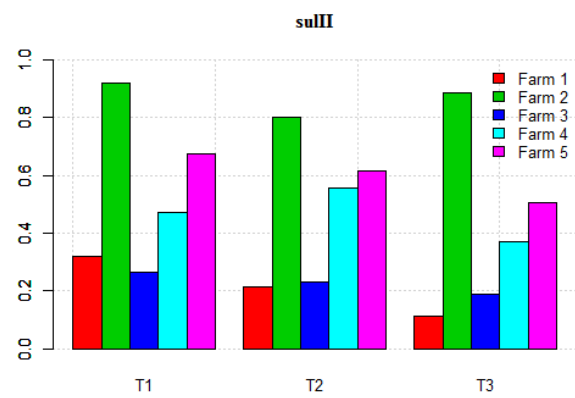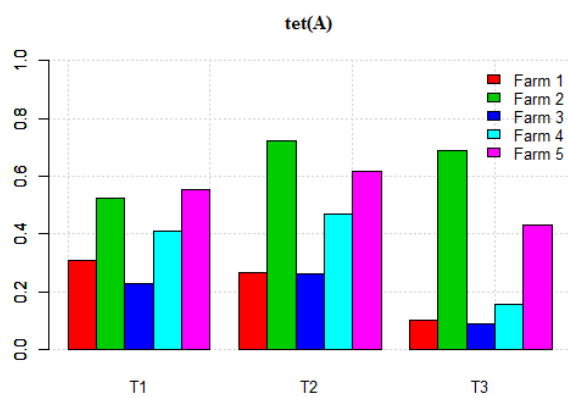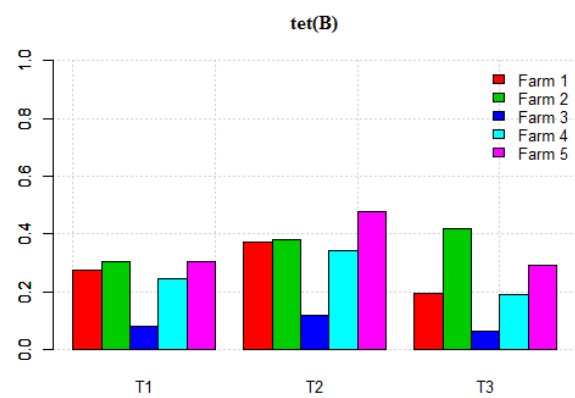

44

45 **FIG S7:** Prevalence of *sulI*, *sulII*, *tet(A)*, and *tet(B)*. Stratified by sampling time and farm.

Supplement: Supplementary file 7 — Additional file 7: Figure S7. Prevalence of sulI, sulII, tet(A), and tet(B). Stratified by sampling time and farm. [file 12866_2019_1619_MOESM7_ESM.pdf]

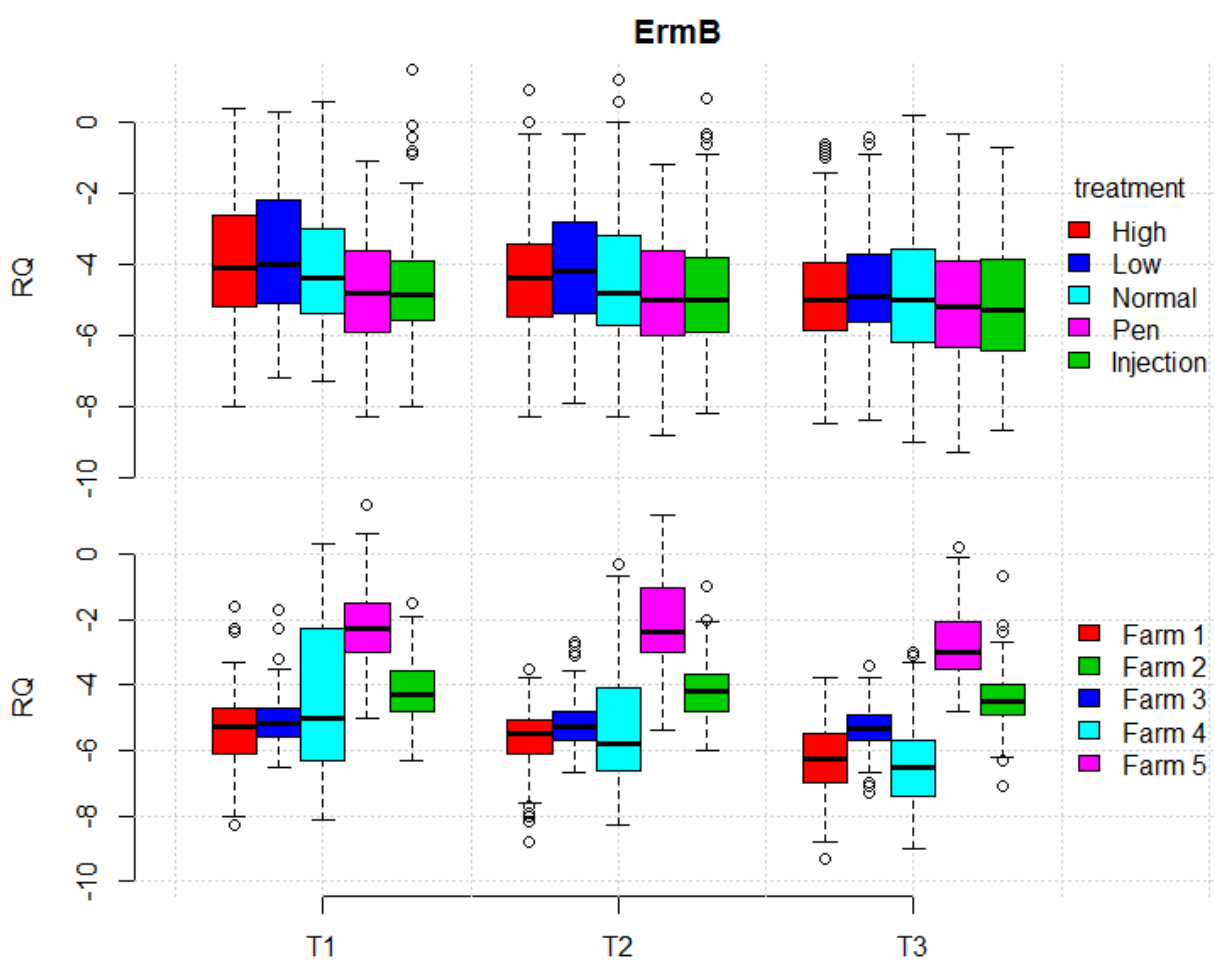

46

47 **FIG S8:** Relative quantities of *ermB* at times T1, T2 and T3. Stratified by treatment and farm.

Supplement: Supplementary file 8 — Additional file 8: Figure S8. Relative quantities of ermB at times T1, T2 and T3. Stratified by treatment and farm. [file 12866_2019_1619_MOESM8_ESM.pdf]

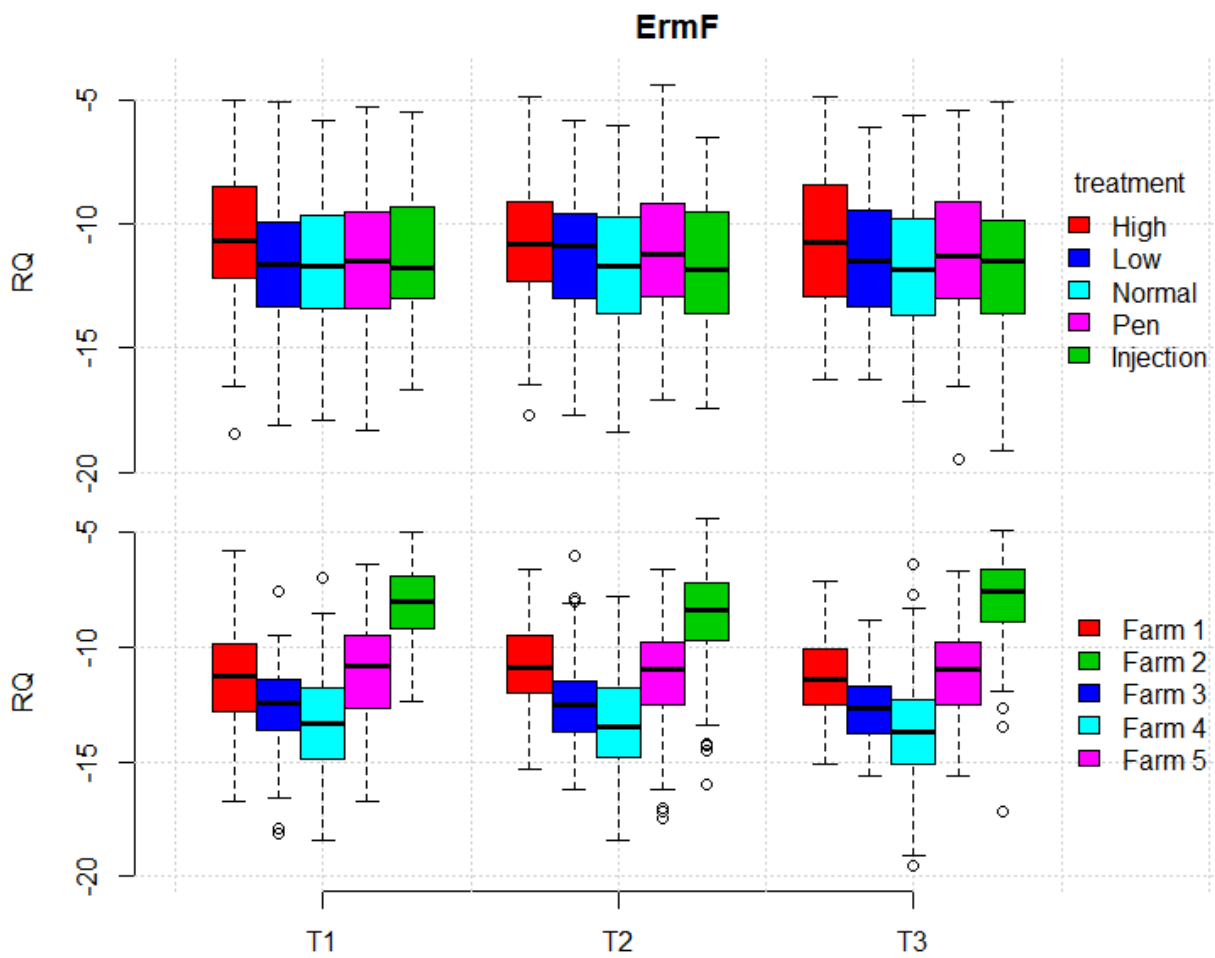

48

49 **FIG S9:** Relative quantities of *ermF* at times T1, T2 and T3. Stratified by treatment and farm.

Supplement: Supplementary file 9 — Additional file 9: Figure S9. Relative quantities of ermF at times T1, T2 and T3. Stratified by treatment and farm. [file 12866_2019_1619_MOESM9_ESM.pdf]

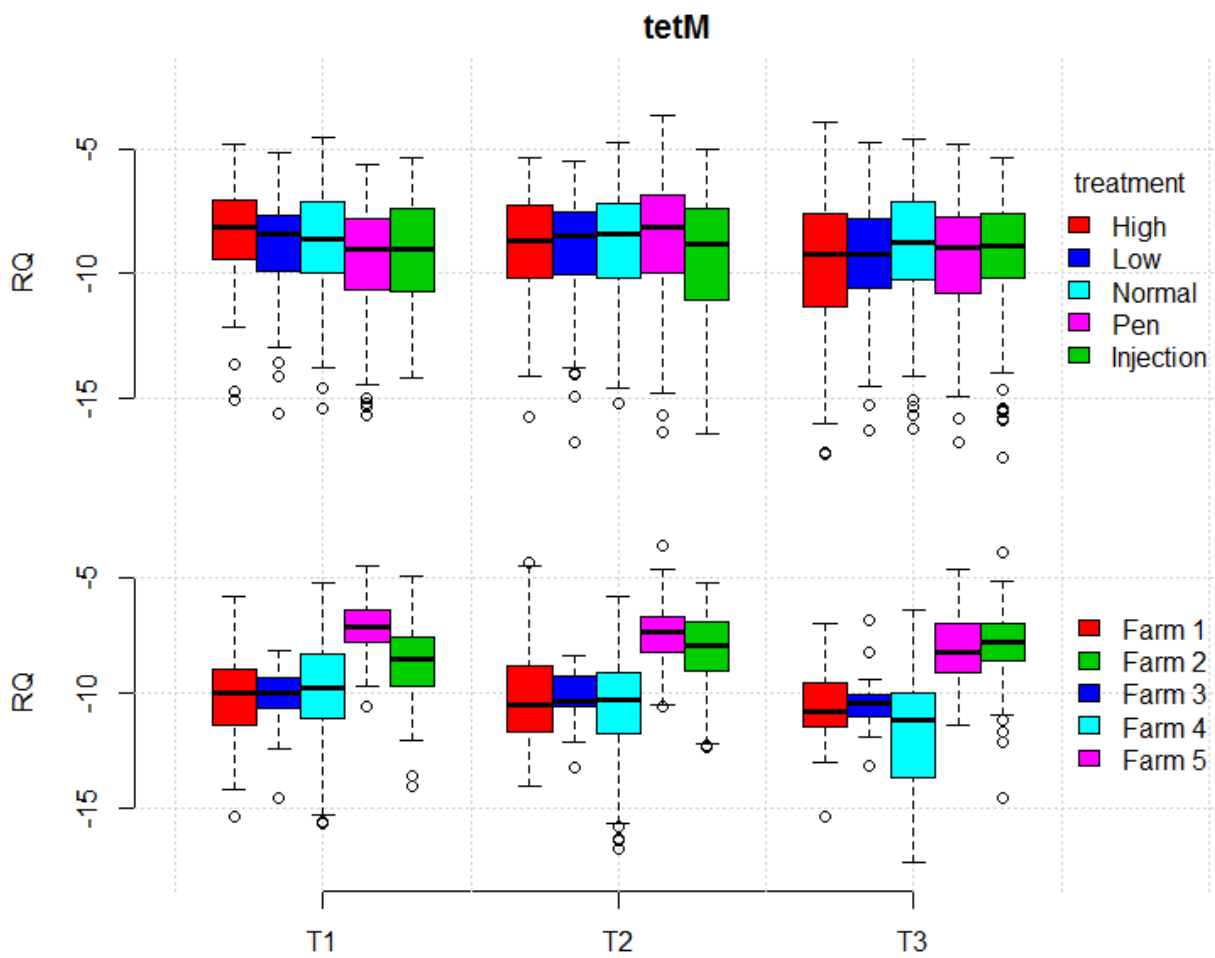

**FIG S10:** Relative quantities of *tetM* at times T1, T2 and T3. Stratified by treatment and farm.

Supplement: Supplementary file 10 — Additional file 10: Figure S10. Relative quantities of tetM at times T1, T2 and T3. Stratified by treatment and farm. [file 12866_2019_1619_MOESM10_ESM.pdf]

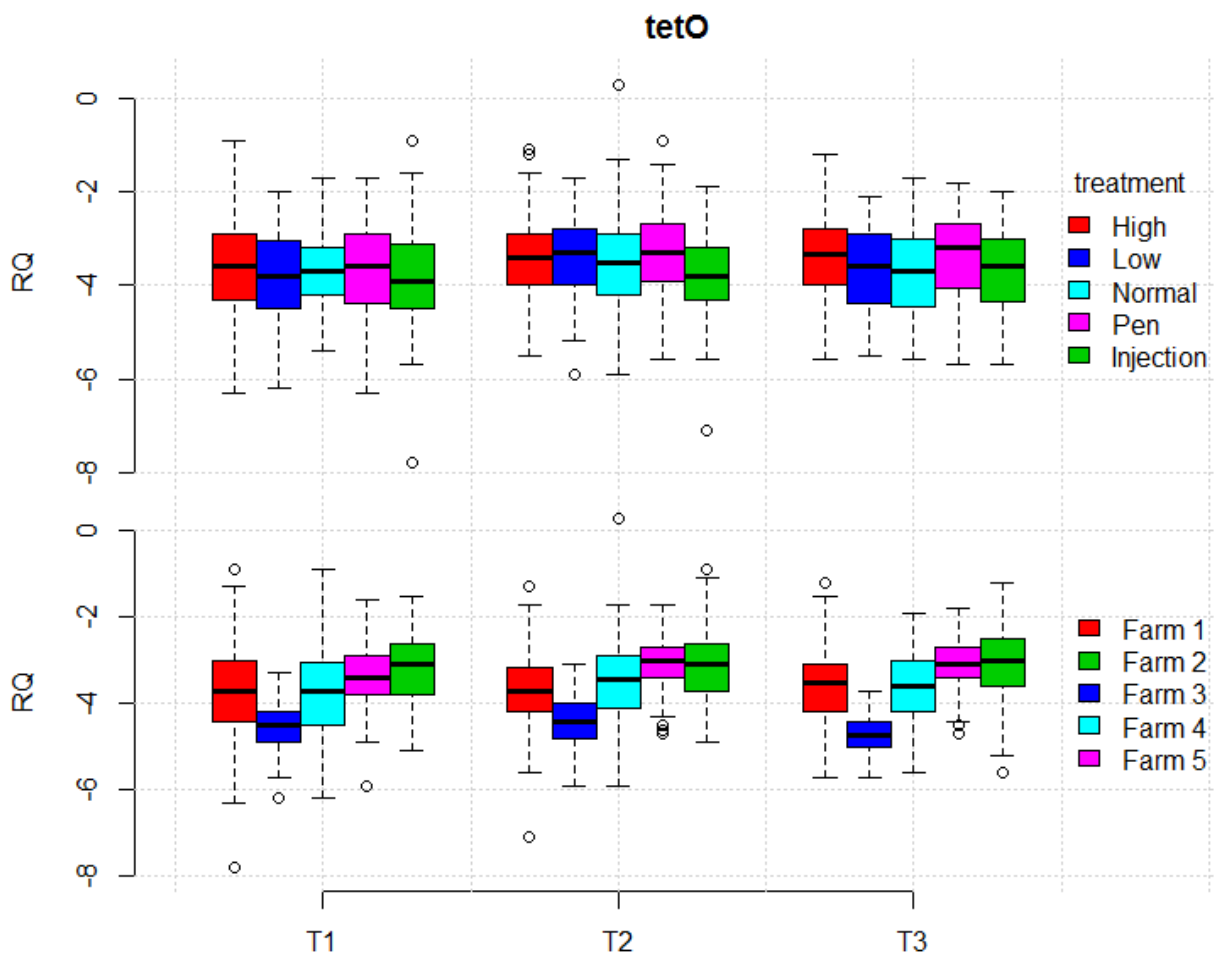

**FIG S11:** Relative quantities of *tet(O)* at times T1, T2 and T3. Stratified by treatment and farm.

Supplement: Supplementary file 11 — Additional file 11: Figure S11. Relative quantities of tet(O) at times T1, T2 and T3. Stratified by treatment and farm. [file 12866_2019_1619_MOESM11_ESM.pdf]

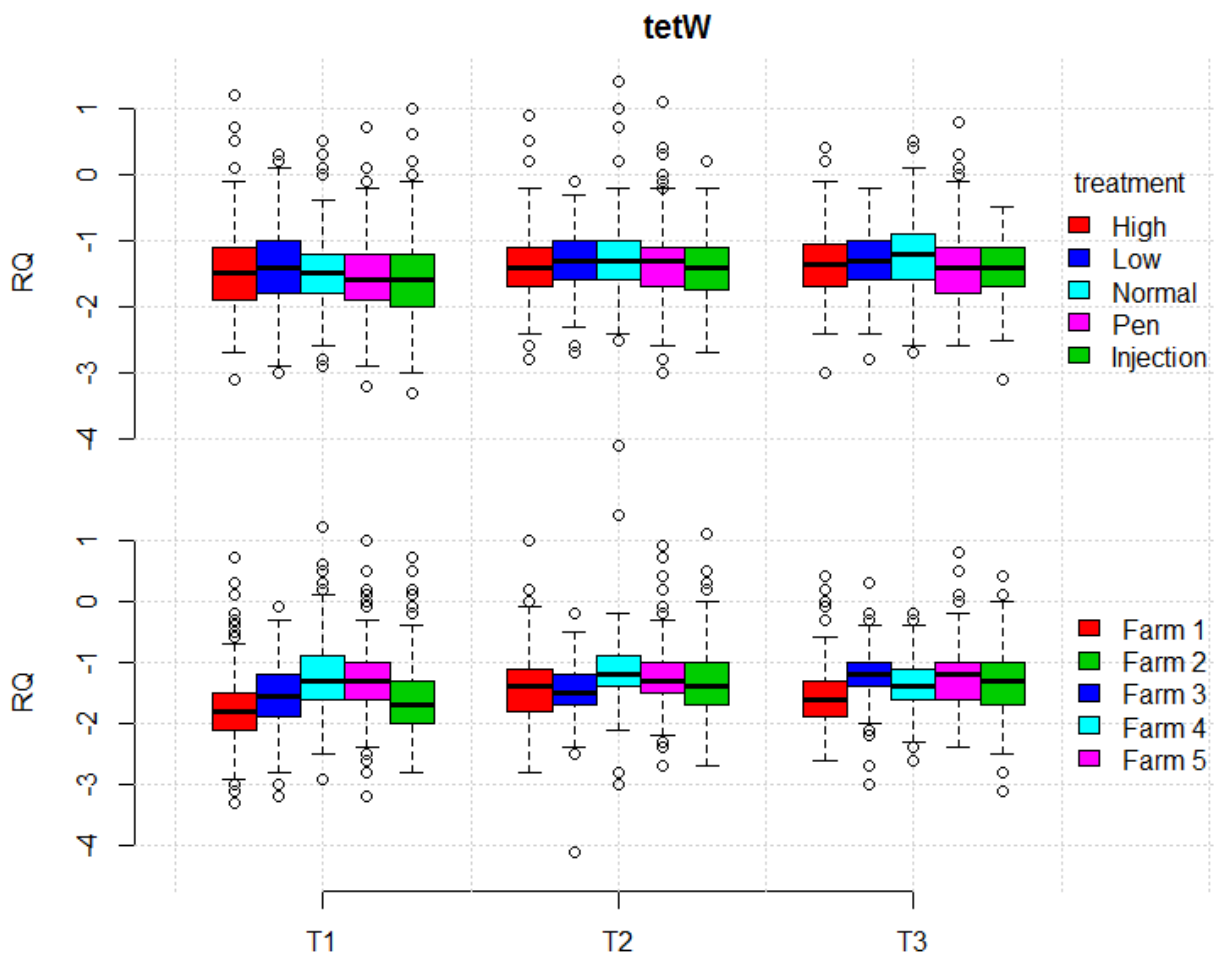

57

58 **FIG S12:** Relative quantities of *tetW* at times T1, T2 and T3. Stratified by treatment and farm.

59

Supplement: Supplementary file 12 — Additional file 12: Figure S12. Relative quantities of tet(W) at times T1, T2 and T3. Stratified by treatment and farm. [file 12866_2019_1619_MOESM12_ESM.pdf]
