# Supplementary material for: Effect of tetracycline treatment regimens on antibiotic resistance gene selection over time in nursery pigs
Source: BMC Microbiol. 2019 Dec 2;19:269. doi: 10.1186/s12866-019-1619-z (PMC6889206; doi:10.1186/s12866-019-1619-z)
Supplement: Supplementary file 16 — Additional file 16: Figure S16. Prevalence of tet(A), tet(B), sulI and sulII of all batches chronologically on farm 4. Top graph is values prior to treatment (T1), middle graph values two days after treatment T2), and bottom graph is values at exit from nursery unit (T3). Dots are median values of each batch. Lines are smoothed values of the data points. [file 12866_2019_1619_MOESM16_ESM.pdf]

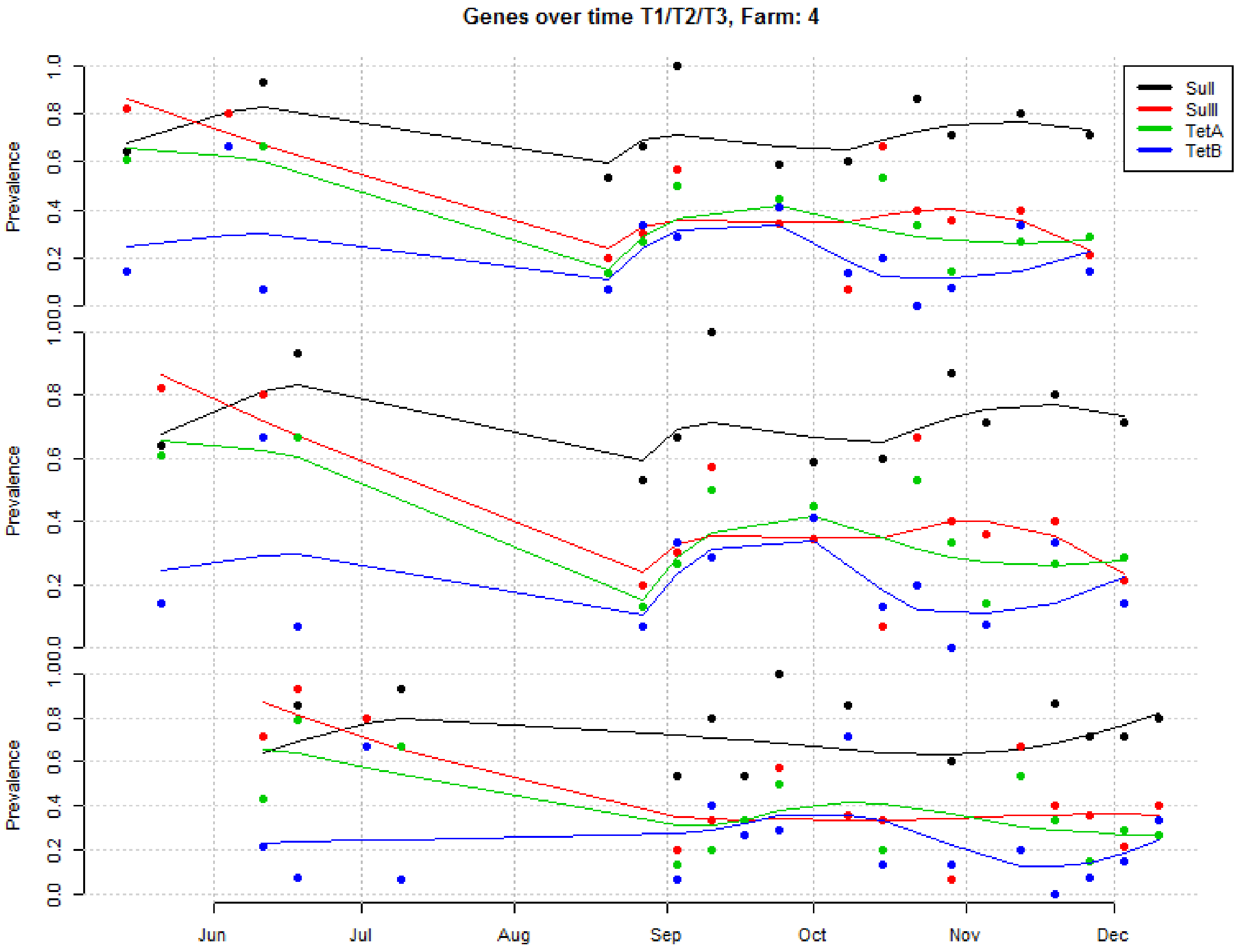

FIG S16: Prevalence of *tet(A)*, *tet(B)*, *sulI* and *sulII* of all batches chronologically on farm 4. Top graph is values prior to treatment (T1), middle graph values two days after treatment (T2), and bottom graph is values at exit from nursery unit (T3). Dots are median values of each batch. Lines are smoothed values of the data points.
