# Supplementary material for: Effect of tetracycline treatment regimens on antibiotic resistance gene selection over time in nursery pigs
Source: BMC Microbiol. 2019 Dec 2;19:269. doi: 10.1186/s12866-019-1619-z (PMC6889206; doi:10.1186/s12866-019-1619-z)
Supplement: Supplementary file 17 — Additional file 17: Figure S17. Prevalence of tet(A), tet(B), sulI and sulII of all batches chronologically on farm 5. Top graph is values prior to treatment (T1), middle graph values two days after treatment (T2), and bottom graph is values at exit from nursery unit (T3). Dots are median values of each batch. Lines are smoothed values of the data points. [file 12866_2019_1619_MOESM17_ESM.pdf]

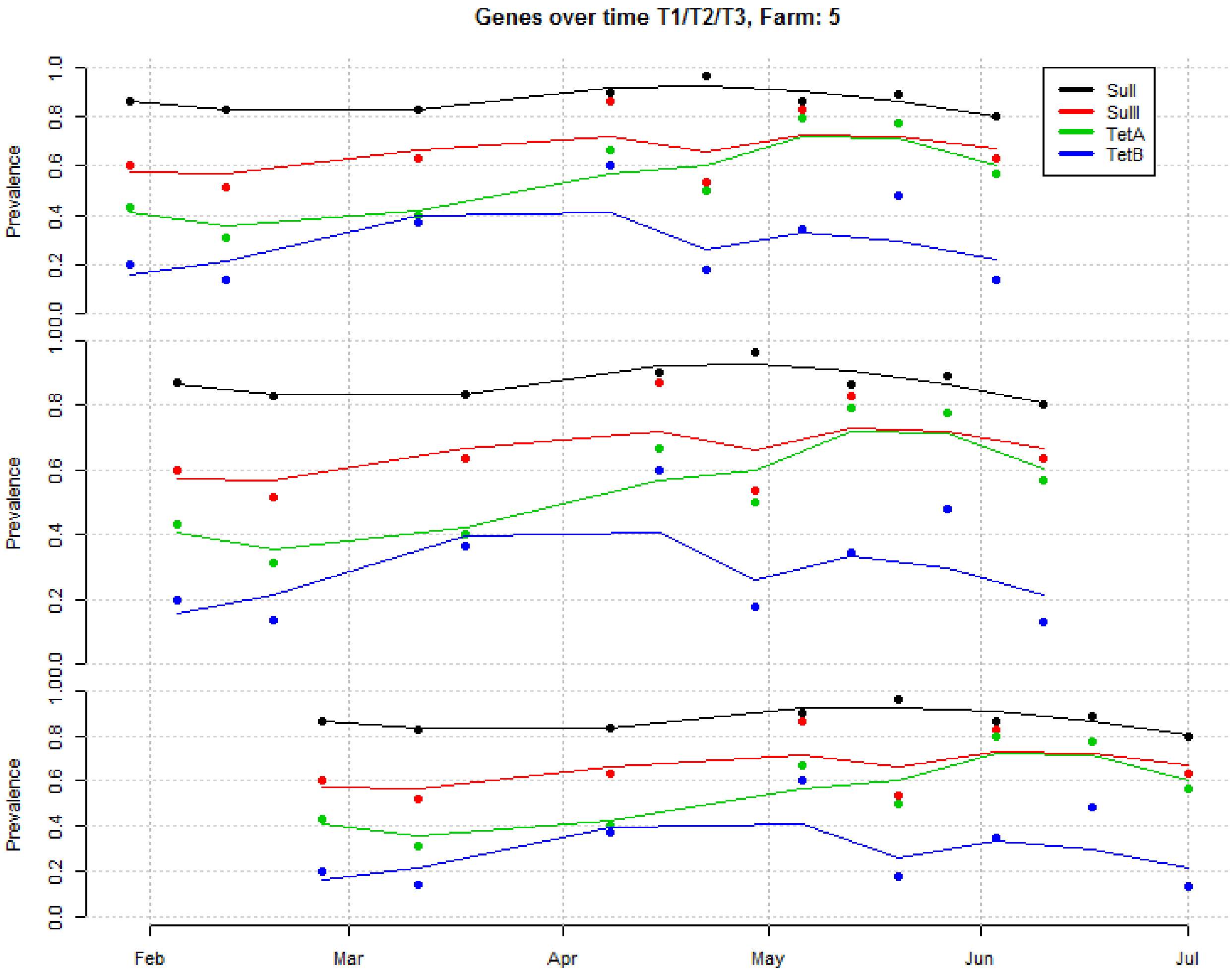

FIG S17: Prevalence of *tet(A)*, *tet(B)*, *sulI* and *sulII* of all batches chronologically on farm 5. Top graph is values prior to treatment (T1), middle graph values two days after treatment (T2), and bottom graph is values at exit from nursery unit (T3). Dots are median values of each batch. Lines are smoothed values of the data points.

References

1. **Bockelmann, U., H. H. Dorries, M. N. Ayuso-Gabella, M. Salgot de Marcay, V. Tandoi, C. Levantesi, C. Masciopinto, E. Van Houtte, U. Szewzyk, T. Wintgens, and E. Grohmann.** 2009. Quantitative PCR monitoring of antibiotic resistance genes and bacterial pathogens in three European artificial groundwater recharge systems. Appl Environ Microbiol **75:**154-63.
2. **Clasen, J., A. Mellerup, J. E. Olsen, Ø. Angen, A. Folkesson, T. Halasa, N. Toft, and A. C. Birkegård.** 2016. Determining the optimal number of individual samples to pool for quantification of average
