# Supplementary material for: Effect of tetracycline treatment regimens on antibiotic resistance gene selection over time in nursery pigs
Source: BMC Microbiol. 2019 Dec 2;19:269. doi: 10.1186/s12866-019-1619-z (PMC6889206; doi:10.1186/s12866-019-1619-z)
Supplement: Supplementary file 18 — Additional file 18: Table S1. [file 12866_2019_1619_MOESM18_ESM.pdf]

TABLE S1

Primers	Gene target	Sequence (5' → 3')	Ann. temp. (°C)	Amplicon size (bp)	GenBank access. no.*	Reference
FP_TETA_2<br>RP_TETA_2<br>PR_TETA_2	*tet*(A)	TTGGCATTCTGCATTCACTC<br>GAAGGCAAGCAGGATGTAGC<br>GATCACCGGCCCTGTAGCCG	60<br>60	125	X00006	(3)
FP_TETB<br>RP_TETB<br>PR_TETB_	*tet*(B)	TTACGTGAATTTATTGCTTCGG<br>ATACAGCATCCAAAGCGCAC<br>CGCCGACCAAATCGGTCAGA	60<br>60	206	NE_013365	(3)
FP_TETC_6<br>RP_TETC_6<br>PR_TETC_6	*tet*(C)	GCCAGTCACTATGGCGTGCT<br>CAAGTAGCGAAGCGAGCAGG<br>ACTGTCCGACCGCTTTGGCC	60<br>60	120	EU751613	(3)
FP_TETM_7<br>RP_TETM_7<br>PR_TETM_7	*tet*(M)	CAACGAGGACGGATAATACGC<br>CCATCTTTTGCAGAAATCAGTAGA<br>GGTGAACATCATAGACACGCCAGGA	60<br>60	191	X92947	(3)
FP_TETO<br>RP_TETO<br>PR_TETO_	*tet*(O)	AAGAAAACAGGAGATTCCAAAACG<br>CGAGTCCCCAGATTGTTTTTAGC<br>ACGTTATTTCCCGTTTATCACGGAAGCG	60<br>60	75	AY660531	(1)
FP_TETW<br>RP_TETW<br>PR_TETW	*tet*(W)	GCAGAGCGTGGTTCAGTCT<br>GACACCGTCTGCTTGATGATAAT<br>TTCGGGATAAGCTCTCCGCCGA	60<br>60	66	AJ222769	(4)

FP_SUL1<br>RP_SUL1<br>PR_SUL1	*sulI*	ACGAGATTGTGCGGTTCTTC<br>CCGACTTCAGCTTTTGAAGG<br>ACCGGCTCATCCTCGATCCG	60<br>60	159	EU056266	(3)
FP_SUL2<br>RP_SUL2<br>PR_SUL2	*sulII*	GATATTCGCGGTTTTCCAGA<br>CGCAATGTGATCCATGATGT<br>AAGACGGGCAGGCAGATCGG	60<br>60	141	AY360321	(3)
FP_ERMB_Böck<br>RP_ERMB_Böck**<br>PR_ERMB_Böck	*ermB*	GGATTCTACAAGCGTACCTTGGA<br>TGGCAGCTTAAGCAATTGCT<br>CACTAGGGTTGCTCTTGCACACTCA<br>AGTC	60<br>60	86	AB563188	(1)
FP_ERMF_KNAPP<br>RP_ERMF_KNAPP<br>PR_ERMF_OWN	*ermF*	TCGTTTTACGGGTCAGCACTT<br>CAACCAAAGCTGTGTCGTTT<br>ATATTGGGGCAGGCAAGGGGTT	60<br>60	182	M14730;M17<br>124;<br>M17808;M62<br>487	(3)
FW_Clasen<br>R_Clasen<br>PR_Clasen	16S rDNA	TGGAGCATGTGGTTTAATTCGA<br>ACTTAACCCAACATTTCAC<br>CACGAGCTGACGACAGCC	60	126	NA	(2)

Forward primer=FP; Reverse primer=RP; Probe=PR, gene targets, Ann.temp = annealing temperatures, amplicon length in basepairs (bp) and GenGenk sequence accession number (Genbank Acces. No.) All probes were labeled with 5’FAM and 3’TAMRA
